# Functional Integration of Newborn Neurons in the Zebrafish Optic Tectum

**DOI:** 10.3389/fcell.2019.00057

**Published:** 2019-04-16

**Authors:** Jonathan Boulanger-Weill, Germán Sumbre

**Affiliations:** ^1^Department of Molecular and Cellular Biology, Center for Brain Science, Harvard University, Cambridge, MA, United States; ^2^Institut de Biologie de l’ENS (IBENS), Département de Biologie, École Normale Supérieure, CNRS, INSERM, Université PSL, Paris, France

**Keywords:** neurogenesis, newborn neurons, zebrafish, activity-dependent development, optic tectum, visual system

## Abstract

Neurogenesis persists during adulthood in restricted parts of the vertebrate brain. In the optic tectum (OT) of the zebrafish larva, newborn neurons are continuously added and contribute to visual information processing. Recent studies have started to describe the functional development and fate of newborn neurons in the OT. Like the mammalian brain, newborn neurons in the OT require sensory inputs for their integration into local networks and survival. Recent findings suggest that the functional development of newborn neurons requires both activity-dependent and hard-wired mechanisms for proper circuit integration. Here, we review these findings and argue that the study of neurogenesis in non-mammalian species will help elucidate the general mechanisms of circuit assembly following neurogenesis.

## Introduction

Neurogenesis is the process by which new neurons are generated from neural progenitor cells. This process starts during embryonic development, where an initial scaffold is generated and populated by massive waves of newborn neurons. In mammals, this process occurs primarily before birth (Götz and Huttner, 2005), except for two brain regions in which neurogenesis remains active during adulthood: the subgranular zone of the dentate gyrus in the hippocampus and the subventricular zone of the lateral ventricles (Ernst and Frisén, 2015). In mice, embryonic neurogenesis begins with the transformation of neuroepithelial cells (around E10) that are located in the ventricular zone and subventricular zone into radial glial cells. Radial glial cells are neuronal progenitor cells that can generate neurons by symmetric (forming two neurons) or asymmetric division (forming a radial glial cell and a neuron), or by the production of intermediate progenitor cells that later undergo symmetric neurogenic divisions (Götz and Huttner, 2005; Yao et al., 2016). Neurons then migrate, acquire electrical excitability, neurotransmitter identity, and develop axons and dendrites (Spitzer, 2006). Eventually, embryonic neurogenesis sets the global morphology of the nervous system. After birth and during adolescence, extensive axonal, dendritic and synaptic pruning is achieved by a phagocytic-dependent process (Riccomagno and Kolodkin, 2015). In humans, this process is likely to be protracted as synaptic elimination continues in the prefrontal cortex until early adulthood (Rakic et al., 2011).

Adult neurogenesis niches contain quiescent radial glia-like neural stem cells that generate intermediate progenitor cells capable of producing neuroblasts. In mice, approximately 30,000 neuroblasts migrate along the rostral migratory stream every day and populate the olfactory bulb, where they differentiate into interneurons (Alvarez-Buylla et al., 2001). Once mature, they participate in olfactory discrimination, and olfactory short- and long-term associative memory (Lepousez et al., 2015). In the hippocampus, ∼9,000 neuroblasts differentiate daily into granule cells and populate the hippocampus (Cameron and Mckay, 2001). To populate the already-functional neuronal scaffold, newborn neurons compete with the existing circuitry to integrate into the network (Toni et al., 2007, 2008; McAvoy et al., 2016). This integration process lasts ∼8 weeks and plays a critical role in long-term spatial learning (Kee et al., 2007), pattern separation (which permits discrimination between two similar inputs) and affective behaviors (Kempermann et al., 2015).

Zebrafish neurogenesis has traditionally been separated into primary and secondary neurogenesis (Chapouton and Bally-cuif, 2004). Primary neurogenesis takes place before 2 days post-fertilization (dpf), and generates a scaffold of large and mostly transient neurons that mediate spontaneous coils and reflexive motor responses to touch stimuli (Kimmel et al., 1991; Wullimann, 2009). These initial neurons pioneer the major brain components and axon tracts of the embryo (Korzh et al., 1993; Chapouton and Bally-cuif, 2004). Secondary neurogenesis, also termed “post-embryonic,” becomes dominant in the hatching larva (Mueller and Wullimann, 2003; Wullimann, 2009) and massively adds neurons to the initial scaffold. At 4 dpf, the nervous system is already mature and the larva already displays a large repertoire of visually induced behaviors (Muto and Kawakami, 2013). Newborn neurons are then added to this functional scaffold in a fashion similar to mammals: neuroepithelial progenitors generate radial glia that serve as progenitor cells with life-long neurogenic potential (Dirian et al., 2014; Galant et al., 2016). In teleosts, up to 16 neurogenesis regions have been described, conferring widespread brain regenerative potential. This capacity may reflect a growing demand of sensory-input processing associated with lifelong body growth (Grandel and Brand, 2013). These qualities make zebrafish an emerging complementary model for the study of newborn neuron integration into mature circuits (Boulanger-Weill et al., 2017; Hall and Tropepe, 2018b).

The optic tectum (OT) has provided important insights into the initial development of post-embryonic neurons (Niell and Smith, 2005; Zhang et al., 2011; Avitan et al., 2017; Pietri et al., 2017). At 5 dpf, the larva is capable of engaging in tectum-dependent complex behaviors such as prey capture (Muto and Kawakami, 2013), and tectal neurons are already responsive to visual stimuli of specific sizes, orientations, and directions (Hunter et al., 2013; Barker and Baier, 2015; Dunn et al., 2016). Due to the small size of the zebrafish larva, its transparency and its genetic accessibility, calcium dynamics of both newborn and mature neurons in the OT can be recorded simultaneously using two-photon microscopy (Boulanger-Weill et al., 2017). These characteristics make the zebrafish OT a complementary and advantageous model to study the mechanisms underlying the incorporation of newborn neurons into the pre-existing circuitry.

Here, we describe the neurogenic niche of the OT and present recent findings on the functional maturation, integration and survival of newborn tectal neurons (Boulanger-Weill et al., 2017; Hall and Tropepe, 2018b). When possible, we provide a comparative description with mammalian neurogenesis. These new studies highlight the power of the zebrafish larva for neurogenesis research and open up exciting new avenues to gain further insights on the general principles underlying the incorporation of newborn neurons into established neuronal circuits.

## Neurogenesis and Cell Diversity in the Zebrafish Optic Tectum

**FIGURE 1:**
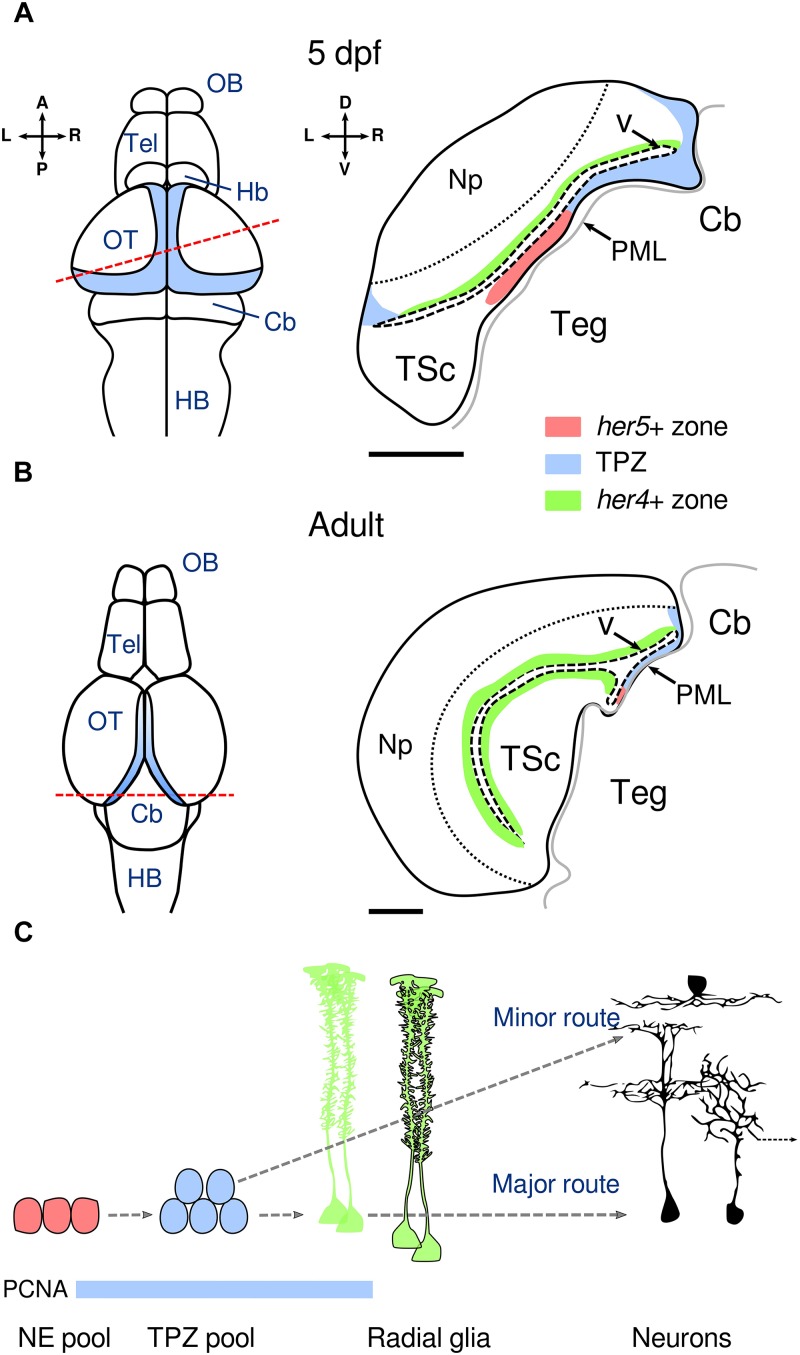
Neuroepithelial neurogenesis in the post-embryonic OT. **(A)** Organization of the neurogenesis niche in the OT at 5 dpf. Left panel: dorsal view of the brain representing the main sub-divisions. The blue shaded region represents the TPZ. The dashed red line indicates the cross-section plane in the right panel. Right panel: schematic cross-section of one OT hemisphere showing in red the neuroepithelial *her5*-positive territory, in blue the TPZ and in green the radial glia *her4*-positive territory. The PML provides the developing tectum with progenitor cells that are progressively pushed upward toward the OT. The thin dashed line represents the separation between the neuropil and the periventricular zone containing most of the neuronal cell bodies. The scale bar represents 50 μm. **(B)** Organization of the neurogenesis niche in the OT in the adult zebrafish (older than 3 months). Left panel: dorsal view of the brain representing the main sub-divisions. The high-rate proliferation area is depicted in blue according to Ito et al. (2010). Orientations as in **(A)**. Right panel as in **(A)** showing the reduction of the *her5*-positive territory. The scale bar represents 100 μm. **(C)** Neurogenic sequences in the post-embryonic OT. The *her5*-positive neuroepithelial pool divides at low rates and generates progenitors in the TPZ with high mitotic activity, which in turn generate *her4*-positive radial glia (major route) or neurons (minor direct route). Radial glia are transiently neurogenic in the OT (non-neurogenic radial glia are outlined in black). Although both routes contribute to the neuronal lineage, their relative contribution to neuronal diversity and their dynamics in post-embryonic stages are unknown. Three representative neuronal subtypes are shown: a superficial interneuron (top), a peri-ventricular projection neuron (right, output to other brain regions indicated by the dashed arrow) and a bi-stratified interneuron (left). PCNA expression is indicated by the blue bar. Dashed arrows indicate lineage relationships. L: left, R: right, A: anterior, P: posterior, D: dorsal, V: ventral, OB: olfactory bulb, Tel: telencephalon, Hb: habenula, Cb: cerebellum, HB: hindbrain, Np: neuropil, TSc: torus semicircularis, Teg: tegmentum, V: ventricle, PML: peripheral midbrain layer, TPZ: tectal proliferation zone. Adapted with permission from Galant et al. (2016).

At larval stages, the OT neurogenic niche forms a continuous and superficial crescent spanning from its dorso-medial to caudo-lateral margins, where it folds ventrally and connects to the torus semicircularis. This peripheral midbrain layer (PML, Figure 1A) contains a discrete *her5*-positive population arranged as a polarized mono-layer of cuboidal cells bound by tight junctions (Galant et al., 2016). They express markers of apico-basal polarity including zona occludens protein 1 (ZO-1), γ-tubulin and aPKC, typical of neuroepithelial cells, and are mosaically positive for Proliferating Cell Nuclear Antigen (PCNA, a marker of the G1 phase of the cell cycle and indicator of cell proliferation, Ito et al., 2010; Galant et al., 2016). Using an elegant genetic tracing approach from post-embryonic to adult stages, Galant et al. (2016) demonstrated that these progenitors generate a transit amplifying pool at the posterior domain of the OT (TPZ, tectal proliferation zone, Figure 1A,B). The TPZ subsequently gives rise to neurons by two distinct mechanisms: directly (minor route) or through transiently neurogenic *her4*-positive glia (major route, Figure 1C). Such bimodal neurogenic activity seems to be present during early post-embryonic neurogenesis (from 5 dpf) (Galant et al., 2016; Hall and Tropepe, 2018b), but their relative contribution still needs to be assessed. This neuroepithelial neurogenesis is also present in the lateral pallium of zebrafish (Dirian et al., 2014) and closely resembles mammalian embryonic neurogenesis, where neuroepithelial cells are generated first and later give rise to neurogenic glia, eventually becoming the major source for the generation of neurons (Götz and Huttner, 2005). It is noteworthy that neurogenic neuroepithelial cells are absent in the adult mammalian brain, where newborn neurons are formed solely by the division of radial glia-like cells (Bond et al., 2015).

In the OT, newborn periventricular neurons do not migrate but are rather pushed away from the TPZ in a conveyor belt manner (Zupanc et al., 2005; Devès and Bourrat, 2012; Boulanger-Weill et al., 2017), while a small population of reelin-expressing interneurons may actively migrate to the neuropil (Del Bene et al., 2010). In the larva’s OT, most neurons become glutamatergic and GABAergic while a minority becomes cholinergic (Robles et al., 2011). Several morphological types have been observed: non-stratified GABAergic or glutamatergic periventricular interneurons (Robles et al., 2011; Barker and Baier, 2015), bi-stratified GABAergic or glutamatergic interneurons (Gabriel et al., 2012), GABAergic periventricular projection neurons (Robles et al., 2011), GABAergic or glutamatergic superficial interneurons (Del Bene et al., 2010; Preuss et al., 2014) and mono-stratified interneurons (Figure 1C). Taken together, these results suggest that tectal neurons are generated by a protracted post-embryonic mechanism involving neuroepithelial progenitors. Future work will help delineate how these common progenitors give rise to such a diverse neuronal population.

## Functional Maturation of Newborn Neurons

**FIGURE 2:**
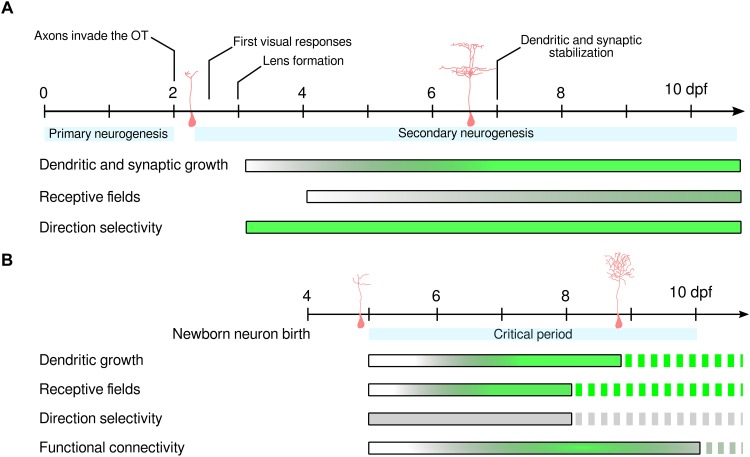
Functional development of OT neurons. **(A)** Maturation time-course of initial tectal neurons from 0 to 10 dpf. After primary neurogenesis has set the initial neuronal scaffold, retinal axons invade the OT, the first visual responses in tectal neurons are observed and the eye’s lens starts forming. Then, secondary neurogenesis continuously supplies the OT with newborn neurons. Neurons born in the early phase of secondary neurogenesis show rapid structural development, acquiring mature dendritic arborization and post-synaptic densities in 4 days (Niell et al., 2004). RF development is a longer process requiring the temporal synchronization of inhibitory and excitatory RFs, which is complete at 9 dpf. This refinement persists in juvenile stages with the reduction of RF width at up to ∼18 dpf (Bergmann et al., 2018). Direction selectivity is already present in early tectal responses, indicating that this property does not require any pruning of initial erroneous connections in order to emerge. This suggests that activity-independent and hard-wired mechanisms enable the formation of direction-selective neuronal responses in the OT. **(B)** Maturation time-course of newborn neurons born at 4 dpf. During an initial sensitive period following newborn neuron generation (blue shade, from 5 to 10 dpf), sensory experience is required to promote neuronal survival. During this initial period, retinal inputs are also required for functional integration into local networks. Newborn neurons acquire mature dendritic arborization and receptive fields during the first 4 days of development. However, the newborn neurons studied in Boulanger-Weill et al. (2017) show weak direction selectivity, suggesting either slower functional development or biased labeling of non-direction-selective neurons. Functional connectivity increased until 8 dpf and decreased thereafter, suggesting pruning of connections among nearby neurons with different receptive fields. Dashed bars indicate lack of information in the literature. Shaded bars indicate the maturation time-course. The length of each bar is indicative of the measurements performed in the literature. Gray indicates immature stage while green indicates complete maturation. For **(A)**, complete maturation indicates that no further refinement has been observed. For **(B)**, complete maturation indicates that newborn neurons have developed similar properties as mature tectal neurons. dpf: days post-fertilization.

The transparency of the larva and its rapid development enable monitoring of the morphological and functional maturation of virtually all neurons from the onset of spontaneous and sensory-induced activity (Niell and Smith, 2005; Warp et al., 2012; Pietri et al., 2017), up to several weeks of development (Boulanger-Weill et al., 2017; Bergmann et al., 2018). As the primary neuronal scaffold is established around 2 dpf, it is possible to monitor both the initial functional assembly of the tectum (Niell and Smith, 2005; Pietri et al., 2017) and the addition of newborn neurons into this scaffold, from their differentiation until their maturity (Boulanger-Weill et al., 2017; Hall and Tropepe, 2018b). Already at 66 hours post-fertilization (hpf), soon after the initial innervation of the tectum by retinal ganglion cells (48 hpf) (Stuermer, 1988), the first post-embryonic neurons display visually induced responses (Niell and Smith, 2005; Figure 2A). Surprisingly, retinotopic organization of the visual responses is already evident at 72 hpf, and only slight modifications occur thereafter (up to 9 dpf) (Niell and Smith, 2005). A few direction-selective neurons are also present at 72 hpf, but their population doubles over the course of 6 days. A recent report showed no changes in the tuning curves of direction-selective tectal neurons between 4 and 7 dpf (Nikolaou and Meyer, 2015). These results suggest that direction selectivity is a cardinal property of tectal neurons that is acquired rapidly after innervation by retinal inputs. For a comprehensive review of direction selectivity in the tectum, see Gebhardt et al. (2013).

Neuronal maturation has also been characterized by measuring the development of the visual spatial receptive fields (RFs) of tectal neurons. These RFs represent the area of the visual field of the larva that triggers activity of a given neuron. In Xenopus tadpoles, a distinctive property of mature tectal neurons is the temporal correlation between inhibitory and excitatory inputs, wherein the latter precede the former (Akerman and Cline, 2006). This delay has been suggested to arise from local feed-forward inhibition, and probably serves to enhance the temporal fidelity of visually induced responses, which is vital during prey capture or predator avoidance (Tao and Poo, 2005; Akerman and Cline, 2006). By using whole-cell voltage-clamp recordings (Zhang et al., 2011) demonstrated that the RFs of tectal neurons first undergo a period of growth from 4 to 6 dpf and then refinement from 6 to 9 dpf. During this period, excitatory inputs to tectal neurons first increase and then decrease, while the size of the inhibitory RFs, initially larger, decrease to match those of the excitatory RFs at later stages (Zhang et al., 2011). A more recent study (Bergmann et al., 2018) suggests that RF size continues to decrease at least until 18 dpf, indicating that tectal circuits show a high degree of plasticity at juvenile stages (Figure 2A). This fine-tuning of visual properties is likely to underlie the continuous improvement in prey capture until adulthood (Westphal and O’Malley, 2013). Therefore, RF maturation is a gradual process that extends at least through the first weeks of larval development.

Apart from the constitutive increase in the number of neurons to adapt to the ever-growing organism, post-embryonic neurogenesis might also promote an increase in visual acuity and efficiency of downstream motor transformations. Indeed, together with the reduction of receptive field sizes, the growth of the tectum could provide higher decoding capabilities in terms of object positions (Avitan et al., 2016). To understand how newborn neurons acquire their mature properties, Boulanger-Weill et al. (2017) monitored the development of their receptive fields over the course of 4 consecutive days, from 5 to 8 dpf as they integrate into the existing functional scaffold. At 1 day old (at 5 dpf), newborn neurons were not visually responsive, indicating that at that developmental stage, they either receive no retinal inputs or lack intrinsic excitability. Two-day-old neurons showed weak and highly variable visual responses, and their RFs were not tuned to any specific region of the field of view. Three-day-old neurons had stronger and less variable visual responses, and their spatial receptive fields were beginning to emerge. Just 1 day later (4-day-old newborn neurons), the neurons displayed mature RFs (Figure 2B).

The pair-wise correlations between the spontaneous activity of newborn and mature neurons can be used as a measure of their functional connectivity (Miller et al., 2014; Romano et al., 2015). Analysis of these correlations in the zebrafish OT showed that for the first 2 days of development, newborn neurons were functionally isolated from the neighboring circuitry. Later on, when the neurons reached 3 days old, they displayed correlated activity with mature neurons at close physical distances. Later (at 10 days old), the number of correlations with other neurons in the circuit significantly decreased, probably reflecting a pruning mechanism to remove initially generated erroneous connections between nearby neurons with different visual receptive fields (Figure 2B). This process also takes place in the visual cortex of the developing mouse, in an activity-dependent manner (Rochefort et al., 2009; Van Der Bourg et al., 2017). Our results show that the functional integration of newborn neurons is a rapid process, lasting just 4 days. During this process, newborn neurons first receive retinal input, acquire tuned visual receptive fields and correlate their spontaneous activity with that of neighboring neurons. Since recordings in juvenile animals have been technically challenging, the terminal stage of functional maturation of tectal neurons remains to be assessed. Future studies will shed light on the full time-course of functional maturation in the OT and describe how it is affected by sensory-motor experience.

## Activity-Dependent Maturation and Integration of Newborn Neurons

Activity-dependent mechanisms play a critical role in mammalian embryonic brain development (Ganguly and Poo, 2013; Pratt et al., 2016). In lower vertebrates, genetically programmed mechanisms are more prevalent, enabling the initial formation of functional circuits even in the absence of sensory stimulation (Niell and Smith, 2005; Pietri et al., 2017). However, sensory experience can later prune and fine-tune initially coarse circuits. For example, exposing Xenopus tadpoles to only 4 h of darkness triggered changes in morphology and excitability in inhibitory neurons, which were rescued by subsequent visual stimulation (He et al., 2016). Similarly, temporary dark rearing of zebrafish larvae significantly reduced the long-term ability of the larvae to hunt prey (Avitan et al., 2017 and see Marachlian et al., 2018 for a review).

In the zebrafish OT, spontaneous activity is organized in topographically compact neuronal assemblies, grouping neurons with strong pair-wise correlations and functionally similar properties (e.g., spatial tuning curves). These neuronal assemblies show attractor-like dynamics and are predictive of directional motor behaviors (Romano et al., 2015; Pietri et al., 2017). Under sensory-deprived conditions (enucleations performed just before newborn neurons start to respond to visual stimuli), newborn neurons in the OT of the zebrafish larva failed to generate functional connectivity with mature neighboring neurons (Boulanger-Weill et al., 2017). Despite enucleation, the mature part of the tectal circuitry remained unaffected, as the spatial and temporal correlations between these neurons were similar to non-enucleated control larvae (Romano et al., 2015; Boulanger-Weill et al., 2017). This result supports the necessity of retinal inputs for the integration but not the maintenance of the initial local functional connectivity. Hall and Tropepe (2018b) carefully examined the survival of newborn neurons when larvae were reared in dim light. They first observed that newborn neuron survival was reduced while proliferation of progenitors and terminal differentiation into mature neuronal subtypes remained unaffected. Second, they demonstrated that exposure to dim light during only the first 5 days (5 to 10 dpf, 0 to 5 days after neuronal birth) of newborn neuron development reduced their survival (Figure 2B). Indeed, later exposure to dim light (5 to 10 days after neuronal birth) did not affect neuronal survival, indicating that a critical period constrains neuronal survival in the OT.

## Discussion and Future Directions

In the zebrafish larva, the maturation of newborn neurons and their incorporation takes ∼4 days. In mammals, the maturation process during adult neurogenesis is significantly slower. Indeed, in mice, newborn neurons take around 8 weeks to reach maturity in the olfactory bulb circuit (Livneh et al., 2014). During this maturation process, newborn neurons display a period of increased sensory responsiveness at 4 weeks and then recede to the specificity observed among the resident neurons (Livneh et al., 2014). Newborn hippocampal neurons, similar to embryonic neurons, are first tonically activated by GABA, which is critical for synaptic integration and morphological development (Ge et al., 2006). Later on, after the GABA switch (transition from excitation to inhibition), neurons undergo a transient period of enhanced pre-synaptic excitability (Marín-Burgin et al., 2012). This process might facilitate the strengthening of weak synaptic inputs and therefore facilitate their integration into local networks. These differences are likely to reflect the challenge newborn neurons face when incorporating into adult neuronal circuits, which are probably less plastic than those observed during development. Indeed, in the mammalian hippocampus, newborn neurons compete with the existing circuitry (McAvoy et al., 2016) for sensory inputs, and this enhanced excitability might facilitate their incorporation. In the zebrafish larva, the conveyor belt organization of the OT imposes an order in which newborn neurons are in direct contact with slightly older neurons, thus forming a smooth functional, molecular and morphological maturity gradient. This arrangement may facilitate newborn neurons’ incorporation without an increase in their excitability. Recent advances in connectomics both in mammals and fish (Wanner et al., 2016; Schmidt et al., 2017) will enable probing the developing synaptic connectivity in these areas and reveal the global dynamics of circuit assembly.

In zebrafish, sensory experience plays a critical role in the survival and incorporation of newborn neurons. In mice, survival of newborn neurons in the adult hippocampus and olfactory bulb has long been thought to be an activity-dependent process (Rochefort et al., 2002; Song et al., 2013; Alvarez et al., 2016). However, a recent report has suggested that under normal conditions, cell death is absent in the olfactory bulb. They suggest that the cell death observed in previous studies was instead induced by the toxicity of the BrdU labeling (Platel et al., 2018). Still, other studies support that acquisition of functional properties and subsequent integration are constrained by sensory inputs in both regions (olfactory bulb and hippocampus). In the mouse olfactory bulb, newborn neurons that were exposed to an olfactory-enriched environment during their increased sensitivity period (from 2 to 5 weeks after neuronal birth), displayed enhanced tuning for odors presented during the enrichment (Livneh et al., 2014). The mature neurons only showed these specific responses to odors enriched during their development and not to control odors. These results indicate that newborn neurons can modify their olfactory tuning curves to adapt to environmental conditions. In the mouse hippocampus, physical exercise or exploration of novel environments can also influence the production, maturation, survival and connectivity of adult-born granule cells (Kempermann et al., 1997; Bergami et al., 2015; Alvarez et al., 2016; Trinchero et al., 2017). Indeed, in a recent study, Alvarez et al. (2016) demonstrated that mouse newborn hippocampal neurons are sensitive to an enriched environment during an early critical window lasting 48 h, 9 days after birth (over an 8-week development period to reach maturity). Three weeks after birth, newborn neurons exposed to this environment displayed longer dendrites and spine densities, suggesting quicker maturation and enhanced integration. Taken together, these results suggest that survival and integration of newborn neurons in the mammalian hippocampus, olfactory bulb and zebrafish OT depend on sensory-induced activity. They highlight the existence of critical periods during which the fate of newborn neurons can be modified: enhancing their synaptic integration and morphological maturation (Alvarez et al., 2016), sensory tuning (Livneh et al., 2014), survival (Alvarez et al., 2016; Hall and Tropepe, 2018b) and functional integration (Boulanger-Weill et al., 2017). However, depending on the brain region or animal model, sensory experience may not always affect the development of newborn neurons. For example, in the mammalian developing cortex, the morphology and migration patterns of vasoactive intestinal peptide (VIP)-positive interneurons are not affected when their excitability is altered (De Marco García et al., 2011). Also, in the zebrafish forebrain, neurogenesis is regulated by motor activity but not by visual inputs (Hall and Tropepe, 2018a).

Newborn neurons are constantly added to the vertebrate brain during adulthood. This observation has sparked the idea that endogenous or exogenous newborn neurons could be used to repair damaged or diseased parts of the brain. In a recent work, Falkner et al. (2016) have demonstrated that embryonic neurons injected in the lesioned visual cortex of adult mice can acquire morphology and functional responses that closely match those of the lost neurons. However, these approaches have shown overall minimal improvement in preclinical trials targeting neurodegenerative diseases (see Barker et al., 2018 for review). Therefore, understanding the mechanisms that enable the survival and integration of newborn neurons into already-developed circuits has been a long-standing goal in neurogenesis research. In recent years, functional integration has been assessed by multiple means: electron microscopy of input or output synapses (Toni et al., 2007, 2008), calcium imaging (Marín-Burgin et al., 2012; Boulanger-Weill et al., 2017) and electrophysiology of evoked neuronal responses (Livneh et al., 2014; Alvarez et al., 2016). The zebrafish larva, suitable for the implementation of all these approaches (Gabriel et al., 2012; Wanner et al., 2016) may open the door for a comprehensive and dynamic characterization of the integration of newborn neurons into established circuits.

## Author Contributions

JB-W and GS wrote the manuscript.

## Conflict of Interest Statement

The authors declare that the research was conducted in the absence of any commercial or financial relationships that could be construed as a potential conflict of interest.
